# A Case of Acute Polyarthritis With a Suspected Association to Mycoplasma Infection Without Extrapulmonary Manifestations

**DOI:** 10.7759/cureus.103040

**Published:** 2026-02-05

**Authors:** Yuta Yoshino, Hirotaka Muramatsu

**Affiliations:** 1 Internal Medicine, Saitama Citizens Medical Center, Saitama, JPN

**Keywords:** acute polyarthritis, epidemic surveillance, extrapulmonary manifestation, infectious diseases epidemiology, mycoplasma pneumoniae infection

## Abstract

Mycoplasma pneumonia, an atypical pneumonia that commonly causes community-acquired pneumonia, can induce extrapulmonary manifestations at different sites, such as the central nervous system, skin, and gastrointestinal tract. These extrapulmonary manifestations can present without respiratory symptoms, making the diagnosis challenging. Herein, we report the case of a 26-year-old woman who presented with polyarthritis following fever and gastrointestinal symptoms. At the time of examination, mycoplasma pneumonia had spread in the community, which led to the diagnosis. Her polyarthritis improved one week after the administration of an antibiotic and a non-steroidal anti-inflammatory drug. Understanding the prevalence of infectious diseases in the community can help in the diagnosis of uncommon presentations based on symptoms, such as the acute onset of polyarthritis, that may be caused by an infectious disease.

## Introduction

Mycoplasma pneumonia, one of the most common types of community-acquired pneumonia, is categorized as an atypical pneumonia. *Mycoplasma pneumoniae* is the main pathogen responsible for atypical pneumonia, accounting for 20% of the community-acquired pneumonia cases, and induces acute pneumonia in 3-13% of cases [1,2]. Accordingly, the rapid diagnosis of mycoplasma infection may be critical in clinical practice. Most patients with mycoplasma infection develop mild self-limiting respiratory symptoms, such as a sore throat and cough. Although *M. pneumoniae* commonly causes pneumonia [3], extrapulmonary manifestations have been reported in up to 25% of patients with mycoplasma infections [4]. These patients can present without respiratory symptoms, but the pathogenesis of extrapulmonary manifestations of *M. pneumoniae* is not yet completely understood [5]. While case reports of acute arthritis following *M. pneumoniae* infection are found, no large-scale epidemiological studies indicating its prevalence are available. In most cases of acute arthritis after infection, the pathogen is often identified based on the type and symptoms of the preceding infection. However, diagnostic tests have limitations in accuracy, making it difficult to establish a clear pathogenic relationship.

Narita classified extrapulmonary manifestations associated with *M. pneumoniae* into the following three mechanisms: (1) a direct infection type in which the pathogen is present at the site of inflammation and induces local inflammatory cytokines; (2) an indirect autoimmune response in which the pathogen is not present at the site of inflammation; and (3) a vascular occlusion in which obstruction of blood flow induces local vasculitis or vascular thrombosis [6]. The treatment of extrapulmonary manifestations of mycoplasma infection is not well documented, suggesting that improvements to treatments may still be possible [7]. Here, we report the case of mycoplasma infection with acute polyarthritis in an adult female with fever and gastrointestinal symptoms. This article indicates that acute arthritis without pneumonia associated with *M. pneumoniae* can be a diagnostic pitfall.

## Case presentation

A 26-year-old woman who was previously healthy presented to our hospital with polyarthralgia lasting for the past three days. She had visited a local physician two weeks earlier for abdominal pain. The physician performed an abdominal ultrasound and laboratory testing at the clinic, which revealed no abnormalities. The abdominal pain persisted for an additional week, resulting in watery stools and loss of appetite. She continued to have a fever (maximum body temperature: 38.4 ℃) for a week before her arrival at our hospital with gradually worsening abdominal pain. Walking had become difficult because of sustained polyarthralgia in the left elbow, left knee, and right ankle joints for the past three days. Her vital signs were as follows: temperature of 38.1 ℃; blood pressure of 146/88 mmHg; heart rate of 76 beats/min; respiratory rate of 16 breaths/min, and SpO_2_ of 96% in room air. Physical examination revealed no abdominal tenderness or palpable lymph nodes on the body surface. The left knee joint was swollen and tender, and severe pain that prevented joint extension was present in the left elbow joint. Concomitant symptoms such as urethritis or skin rash were absent.

**Figure 1:**
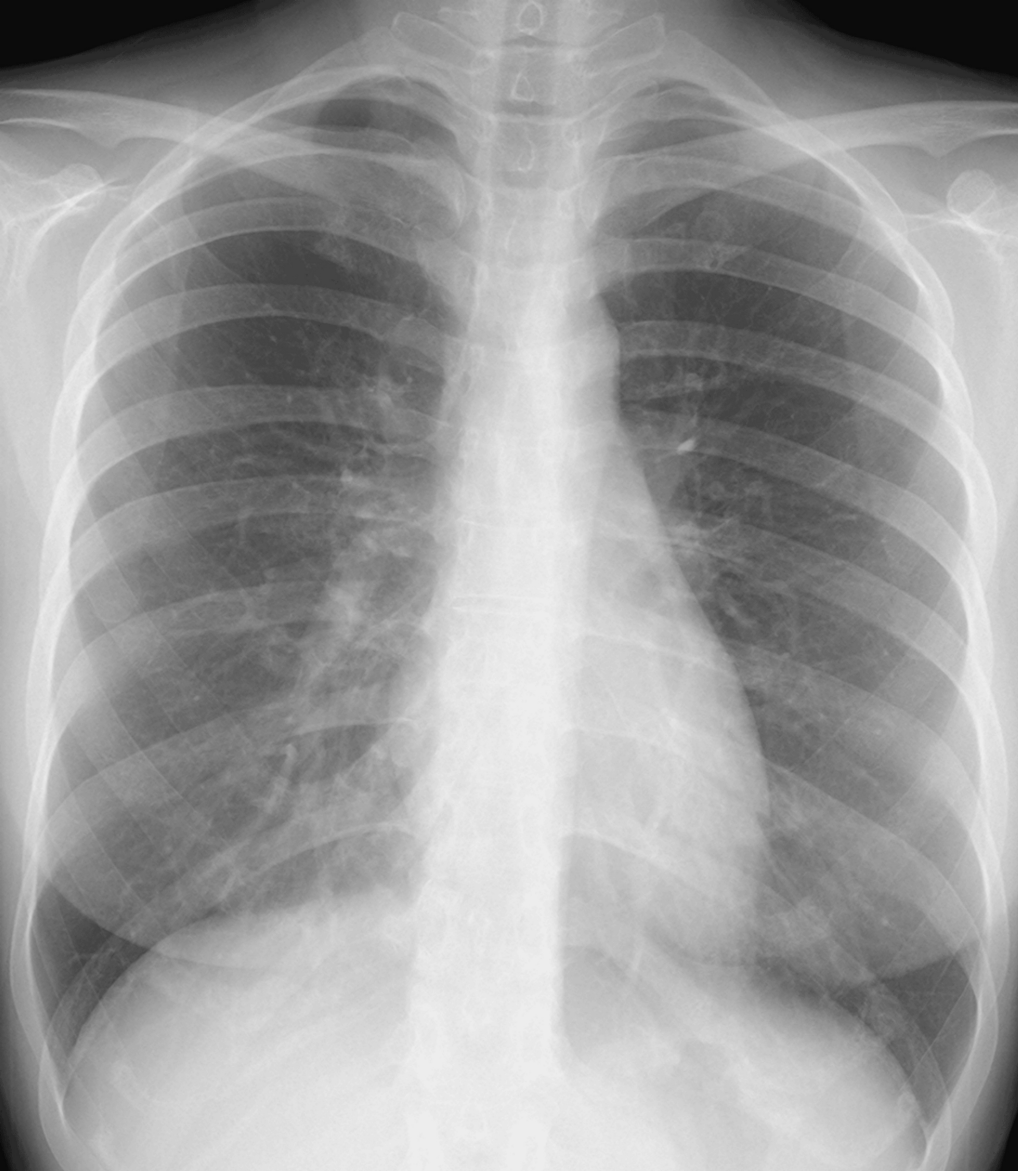
A chest radiography at the initial visit. The chest imaging was a posterior-anterior view. Pneumonia was not found on chest radiography performed at the initial visit.

**Figure 2:**
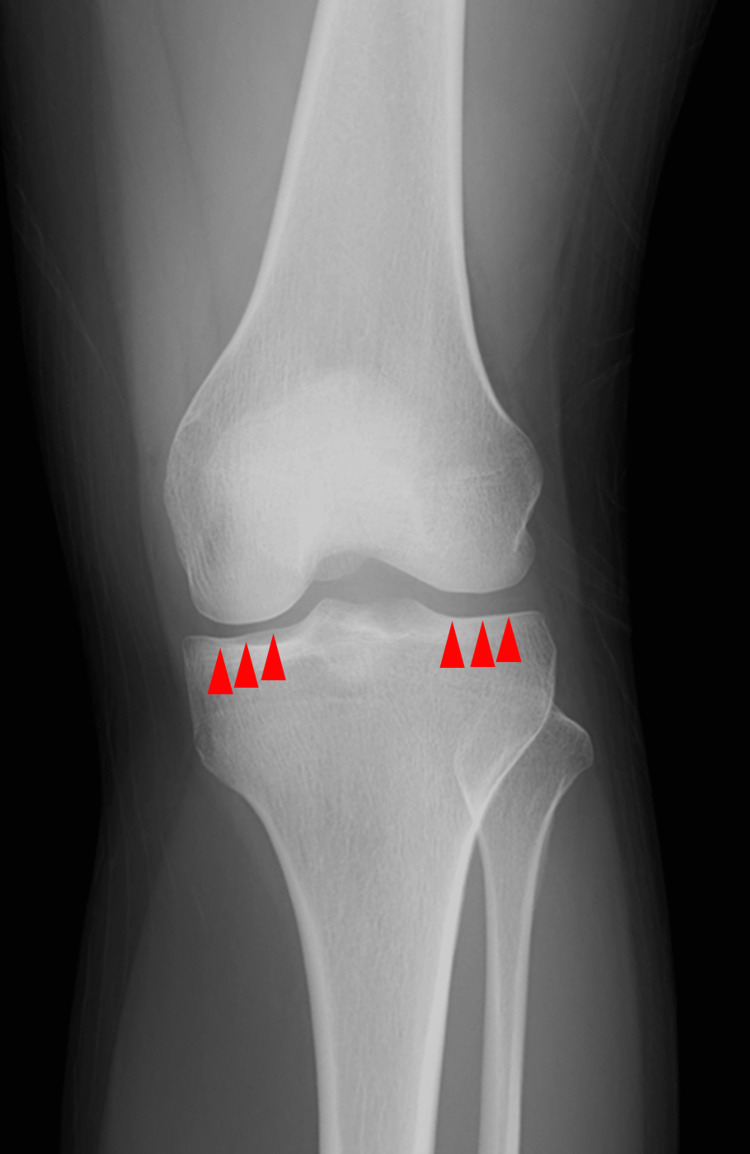
A radiographic testing of the left knee joint. The knee imaging was the anterior-posterior view. Calcinosis suggesting crystal-induced arthritis was not detected on radiographic testing of the left knee joint.

Blood tests performed at the first visit revealed a high inflammatory response, with a C-reactive protein of 4.12 mg/dL, rheumatoid factor level of 12 IU/mL, anti-streptolysin O antibody level of 221 IU/mL, and white blood cell count of 6,900/mm^3^ containing 1% atypical lymphocytes in the leukocyte fraction. Although the patient's left knee joint was swollen, insufficient joint fluid retention had not allowed for the joint cavity puncture. Furthermore, no symptoms were present that would allow for sample collection to detect pathogens other than arthritis. Blood cultures were negative, which reduced the possibility of infectious arthritis as the diagnosis. Furthermore, a stool culture was normal flora, indicating that no pathogens were found in the culture tests that could cause reactive arthritis. Radiography revealed no pneumonia (Figure 1) or calcinosis in the joint space of the left knee (Figure 2). The presence of enthesitis was not evaluated due to the absence of musculoskeletal ultrasound.

*M. pneumoniae* and human parvovirus-B19 were spreading in Japan at the time of this patient's examination. Therefore, reactive arthritis caused by these pathogens was suspected, and serological tests to detect the presence of these pathogens were conducted. From the initial consultation, the patient received loxoprofen and clarithromycin 400 mg per day during follow-up. Three days after the start of treatment, a mycoplasma particle agglutination (PA) titer of 1:320 was observed, and the patient was diagnosed with extrapulmonary symptoms due to mycoplasma infection (Table 1).

**Table 1:** Hematological and biochemical tests. Abbreviations: PA, particle agglutination

Variable	Result	Normal values
White cell count (/mm^3^)	6900	3500–9700
Neutrophils (%)	65.3	42–74
Eosinophils (%)	2.6	0–7
Basophils (%)	0.4	0–2
Monocytes (%)	4.4	1–8
Lymphocytes (%)	26.3	18–50
Atypical lymphocytes (%)	1.0	
Red cell count (*10^6^/µL)	4.94	4.38–5.77
Hemoglobin (g/dL)	14.2	13.6–18.3
Hematocrit (%)	42.1	40.4–51.9
Platelets (*10^4^/µL)	21.2	14.0–37.9
Total bilirubin (mg/dL)	0.6	0.3–1.2
Aspartate aminotransferase (U/L)	15	10–40
Alanine aminotransferase (U/L)	8	5–45
Lactate dehydrogenase (U/L)	185	120–245
Urea nitrogen (mg/dL)	10.3	8–20
Creatinine (mg/dL)	0.53	0.65–1.09
Sodium (mEq/L)	138	135–145
Potassium (mEq/L)	3.5	3.5–5.0
Chloride (mEq/L)	101	98–108
C-reactive protein (mg/dL)	4.12	0–0.3
Rheumatoid factor (IU/mL)	12	<15
Anti-streptolysin O antibody (IU/mL)	221	0–240
Mycoplasma PA titer	1:320	
Human parvovirus-B19 IgM antibody enzyme immunoassay	Negative	Negative
Rapid Plasma Regain test	Negative	Negative
Treponema pallidum hemagglutination test	Negative	Negative
Hepatitis B surface antigen	Negative	Negative
Blood culture	Negative	Negative
Stool culture	Normal flora	Normal flora

At the follow-up examination on the seventh day of treatment, the swelling in the patient's left knee joint had resolved. In addition, pain in the left elbow and right ankle joints reduced. Self-limiting polyarthritis reduces the possibility of septic arthritis and is less likely to be the beginning of chronic polyarthritis, such as rheumatoid arthritis or systemic lupus erythematosus. Paired serum samples for the PA method were not submitted because the extrapulmonary manifestations of the mycoplasma infection showed rapid recovery. Single testing could not determine whether the infection was acute or past. However, the absence of recent infection symptoms and the high PA titer led to a suspected diagnosis of mycoplasma infection with limitations. Treatment with loxoprofen and clarithromycin was also completed for seven days.

## Discussion

In the present case, the patient developed polyarthritis after a fever and gastrointestinal symptoms. The differential diagnosis of acute polyarthritis with fever includes infectious arthritis, post-infectious or reactive arthritis, rheumatoid arthritis, Still's disease, systemic rheumatoid illnesses (e.g., systemic lupus erythematosus), and crystal-induced arthritis [8]. Because the stool culture was negative, it was appropriate to expect that a disease with gastrointestinal symptoms was associated with polyarthritis. The following pathogens are known to cause acute polyarthritis: *Neisseria species*, *Streptococcus pneumoniae*, *Haemophilus influenzae*, Group G Streptococci, and *M. pneumoniae* [9]. Although campylobacter enteritis can induce reactive arthritis, no pathogens were detected in stool cultures. Examination based on the prevalence of infectious diseases led to a suspected association with mycoplasma infection. Although *M. pneumoniae* could not be detected in the joint fluid, the polyarthritis in the present case may potentially be an extrapulmonary manifestation of mycoplasma infection.

*M. pneumoniae* can cause extrapulmonary manifestations independent of respiratory symptoms, with a prevalence of 25-35%, which include symptoms of encephalitis, meningitis, erythema nodosum, and polyarthralgia [2,5,10,11]. Among these extrapulmonary manifestations, gastrointestinal symptoms, such as vomiting, diarrhea, and abdominal pain, were the most common, followed by a cutaneous appearance. Biagi et al. [12] reported that 15 (10.3%) of 145 patients diagnosed with mycoplasma infection had only extrapulmonary manifestations. Gordon et al. [13] reported that 11% of mycoplasma infections presented with extrapulmonary manifestations in the absence of respiratory symptoms. The primary route of infection for *M. pneumoniae* is via the respiratory tract, where the pathogen can potentially pass through the vulnerable intercellular spaces of damaged alveolar epithelium into the systemic circulation [14]. *M. pneumoniae* may cause extrapulmonary manifestations by migrating to distant organs through bacteremia [15]. However, the pathogenesis of extrapulmonary manifestations that develop without respiratory symptoms remains largely unclear [5]. Although extrapulmonary symptoms of mycoplasma infection have been observed in both children and young adults, no characteristic clinical background is available.

Polymerase chain reaction (PCR) and culture of clinical samples collected from the upper respiratory tract using pharyngeal swabs can be inappropriate for the diagnosis of mycoplasma infection without respiratory involvement [5]. Culture tests are not routinely used in clinical practice because isolation of *M. pneumoniae* is expensive, slow, and not widely available. Serological methods used to detect *M. pneumoniae* require the collection of acute and convalescent serum samples two to three weeks apart. At present, the PA test is widely used in Japan for the serological diagnosis of mycoplasma infection, while the enzyme immunoassay is most frequently available worldwide [16]. While the diagnosis of mycoplasma infection can be made when the PA titer is greater than 1:320 in a single serum sample [2], the possibility of false positives in PA testing is a diagnostic limitation in this case. PCR is a useful diagnostic tool for detecting *M. pneumoniae* at the extrapulmonary site because of its rapidity and high sensitivity [4]. PCR of cerebrospinal fluid can be used to diagnose extrapulmonary manifestations of *M. pneumoniae* complications in the central nervous system [17,18]. If the patient's swollen left knee joint in the present case had been punctured, PCR testing of the joint fluid could have been used to detect *M. pneumoniae*. However, serological testing should always be performed because it is difficult to distinguish between acute and persistent infections using PCR [19].

Nonspecific myalgia, arthralgia, and polyarthritis were found in 14% of the extrapulmonary manifestations of mycoplasma infection [20]. In the present case, the arthritis resolved completely as naturally expected. Since evidence for the treatment of extrapulmonary manifestations due to *M. pneumoniae* is insufficient [7], standard care for persistent symptoms has not been established. Although corticosteroids and immunoglobulins have been shown to be beneficial in the treatment of the most severe extrapulmonary manifestations, such as encephalitis and Stevens-Johnson syndrome [21], the evidence for these therapies has been limited to case reports and case series. Based on the pathogenesis of extrapulmonary manifestations and the response to corticosteroids, the concomitant use of antibiotics with immunomodulators can be effective for the treatment of extrapulmonary manifestations [16].

Mycoplasma infections frequently increase during the winter and fall. Historically, epidemics of *M. pneumoniae* have occurred every three to seven years worldwide [22]. In the United States, epidemics of mycoplasma infections tend to occur in late summer and early fall [23], with a similar trend observed in Italy and other regions with warmer climates [24]. However, mycoplasma infections reportedly occur throughout the year, although seasonality of the epidemic has been shown [10]. In Japan, in 2024, the number of mycoplasma infections began to increase in June and continued to increase until November [25], with the number of infections peaking in epidemiologic week 46 of 2024, significantly exceeding the average for the same period over the past five years. In the present case, the onset of polyarthritis coincided with the nationwide peak of the *M. pneumoniae* epidemic in Japan.

Extrapulmonary manifestations of *M. pneumoniae* are nonspecific and can develop without any obvious clinical or imaging features of mycoplasma infection. Therefore, diagnosis is challenging in the early stages of infection, and its symptoms are likely to be underestimated [26]. Even in the absence of respiratory symptoms, mycoplasma infection should be considered in patients with unexplained systemic inflammation. If infectious disease involvement is suspected when the focus of the symptoms is not defined, the local epidemic surveillance of circulating pathogens may be helpful in making a diagnosis.

## Conclusions

*M. pneumoniae* can present with extrapulmonary manifestations without respiratory symptoms. Even in the absence of respiratory symptoms, mycoplasma infection should be considered as a differential diagnosis in patients with unexplained systemic inflammation. In *Mycoplasma*-associated arthritis, the diagnosis depends on the paired serum PA testing and PCR, making it difficult to definitively detect the pathogen with available tests. Understanding the local epidemic surveillance of infectious diseases may be helpful for diagnosing pathogens.
